# Cancer Treated with Carbolic Acid

**Published:** 1872-11

**Authors:** 


### CANCER TREATED WITH CARBOLIC ACID.

The *American Journal of Medical Sciences* contains a report from Surgeon Bill, U. S. Army, of four cases illustrative of this treatment. Two of them were "cured," and the fourth was uuder treatment at the time of the report.

1st Case. An old man; had had labial epithelioma previously removed, and the disease returned in the citatrix and could not be removed. Sixteen grains of acid daily for forty-nine days. Disease had then completely disappeared. That was three years ago, and no indications of a return are at present visible.

2d Case. Similar in situation to the foregoing. Had been excised twice. After its third appearance, carbolic acid, fifteen grains daily, internally, was given. Disease disappeared, and no return noticed thereafter.

Dr. Bill thinks the acid ought to be diluted freely—three grains to the ounce of water.—*Chicago Medical Journal.*

